# Supplementary material for: Brain activity response to cues during gait in Parkinson’s disease: A study protocol
Source: PLoS One. 2022 Nov 17;17(11):e0275894. doi: 10.1371/journal.pone.0275894 (PMC9671304; doi:10.1371/journal.pone.0275894)
Supplement: S2 File — (DOCX) [file pone.0275894.s002.docx]

**Brain activity response to cues during gait in Parkinson's**

**Protocol Version 4.0**

**20^th^ September 2021INVESTIGATORS**

***Principle investigator:***

Dr Sam Stuart, PhD

Vice Chancellors Senior Research Fellow and Honorary Clinical Physiotherapist

Department of Sport, Exercise and Rehabilitation, Northumbria University, Northumberland Building (NB318), Newcastle upon Tyne, NE1 8ST

🕿 0191 227 3343

e-mail: sam.stuart@northumbria.ac.uk

***Co-investigators:***

Dr Rosie Morris, PhD

Senior Lecturer in Neurological Physiotherapy and Honorary Clinical Physiotherapist

Department of Sport, Exercise and Rehabilitation, Northumbria University, Northumberland Building (NB318), Newcastle upon Tyne, NE1 8ST

🕿 0191 237 3343

e-mail: [rosie.e.morris@northumbria.ac.uk](mailto:rosie.e.morris@northumbria.ac.uk)

Julia Das

PhD Candidate and Honorary Clinical Physiotherapist

Department of Sport, Exercise and Rehabilitation, Northumbria University, Northumberland Building (NB318), Newcastle upon Tyne, NE1 8ST

🕿 0191 227 3343

e-mail: [Julia.das@northumbria.ac.uk](mailto:Julia.das@northumbria.ac.uk)

Assistant Professor Martina Mancini, PhD

Assistant Professor of Neurology and Biomedical Engineering

Department of Neurology, Oregon Health and Science University, Portland, Oregon, USA, 97239

🕿 +1 503 494 7230

e-mail: [mancinim@ohsu.edu](mailto:mancinim@ohsu.edu)

Professor Richard Walker, MD

Consultant and Honorary Professor of Ageing and International Health

Northumbria Healthcare NHS foundation trust, North Tyneside General Hospital, Rake Lane, North Tyneside, NE29 8NH

🕿 0191 293 2709

e-mail: [Richard.Walker@northumbria-healthcare.nhs.uk](mailto:Richard.Walker@northumbria-healthcare.nhs.uk)

Dr Rodrigo Vitorio

Research Fellow

Department of Sport, Exercise and Rehabilitation, Northumbria University, Northumberland Building (NB318), Newcastle upon Tyne, NE1 8ST

🕿 0191 227 3343

e-mail: [rodrigo.vitorio@northumbria.ac.uk](mailto:rodrigo.vitorio@northumbria.ac.uk)

**Synopsis of Study**

| Study Title | Brain activity response to cues during gait in Parkinson's (BARC-PD) |
| --- | --- |
| Internal ref. no. (or short title) | Brain activity during gait in Parkinson's |
| IRAS Number | 286383 |
| Study Design | Basic science study involving procedures with human participants, with an exploratory observational design. |
| Study Participants | 80 people with Parkinson’s disease   - 20 Hoehn & Yahr stage I (early disease, minimal symptoms) - 30 Hoehn & Yahr stage II (mild disease, no balance issues) - 30 Hoehn & Yahr stage III (moderate disease, balance issues) |
| Planned Size of Sample (if applicable) | 80 |
| Follow up duration (if applicable) | N/A |
| Planned Study Period | 24 months (1^st^ October 2020 to 30^th^ September 2022) |
| Research Question/Aim(s) | Aim I: Determine brain activity response to cue stimulus (visual, auditory or tactile) when walking in PD.  Aim II: Examine changes in brain activity to cues at different stages of PD. |

# FUNDING AND SUPPORT IN KIND

| **FUNDER** | **FINANCIAL AND NON FINANCIALSUPPORT GIVEN** |
| --- | --- |
| **Parkinson’s Foundation**  **Clinical Research Award (PDF-CRA-2073)**  **PI: Dr Samuel Stuart** | **$200,000** |

**Lay Summary**

Walking problems, such as slow and short steps, are very common in Parkinson’s disease and lead to increased falls risk, as well as reduced mobility and quality of life. Walking issues are difficult to treat as medication interventions do not restore walking ability in people with Parkinson’s, therefore physiotherapy approaches are used to help improve walking. Various physiotherapy strategies have been used, such as internal (thinking about bigger steps) or external prompts. External prompts include auditory (a metronome beat to step in time to), visual (lines to step over on the floor) and tactile (metronome-like vibration to step with) prompts that are very commonly used to improve walking in Parkinson’s. However, the reason why walking improves in people with Parkinson’s with these physiotherapy strategies is unknown, which has led to not all patients benefiting and only short-term walking improvements being seen.

The main issues are that it is unclear if these various internal or external prompt strategies are effective with the progression of Parkinson’s disease, and it is unknown which type of strategy is most effective at different disease stages or with more severe walking impairment, such as freezing (the inability to progress walking for short periods despite wanting to do so). Being able to use specific brain regions to pay attention to different internal or external prompts has been suggested to be the reason why people with Parkinson’s can overcome their walking problems, but this has not been tested. Therefore, this study will use state-of-the-art digital technology to measure walking and brain activity changes with different internal and external prompts. We think that the walking improvement with different prompt strategies relies on the ability to activate specific brain regions, and that brain region activity in response to internal or external prompts will change at different stages of Parkinson’s disease.

Ultimately, understanding the reasons why people benefit from these physiotherapy strategies and who benefits most from specific strategies will enable clinicians to provide more timely and efficient treatment for people with Parkinson’s, and to develop more effective strategies to further improve walking.

**Scientific Background**

Gait impairments are a common and early feature of Parkinson’s disease (PD), and are a major cause of functional dependence, falls and death [[1](#_ENREF_1)]. Gait impairments in PD can be continuous or intermittent, with deterioration of gait with disease progression. Continuous impairments have been the focus of most gait-related research in PD and include reduced step length, speed, increased double-support time and a-rhythmicity [[2](#_ENREF_2)]. Intermittent gait deficits include festination and freezing of gait (FOG) [[2](#_ENREF_2)], which involves a failure to initiate or maintain walking. Intermittent deficits are particularly evident in later disease stages (Hoehn & Yahr (H&Y) stages III-V), and are elicited during turning, gait initiation or with greater task complexity such as walking through doorways or navigating obstacles [[3](#_ENREF_3)]. Gait deficits in PD have largely been attributed to reduced dopamine within the nigro-striatal pathway, but this does not fully explain gait changes in PD across disease severity.

There is currently no effective treatment for gait impairment in PD. Dopaminergic medication has limited effect on gait deficits in PD [[4](#_ENREF_4)], with unsatisfactory improvement in selective characteristics, i.e. speed and step length largely improve but deficits do not resolve compared to controls [[5](#_ENREF_5)]. Similarly, recent cholinergic therapy shows promise [[6](#_ENREF_6)] but despite this evidence for pharmalogical intervention for gait impairment remains limited. Similarly, deep brain stimulation (DBS) also has limited impact on gait deficits and can sometimes have a negative effect. Clinical implementation of non-pharmalogical physiotherapy strategies, such as cueing, can help to ameliorate gait issues and reduce falls risk [[7](#_ENREF_7)]. Internal (i.e. thinking about larger steps) and external cue stimulus (e.g. auditory (i.e. metronome beats to step in time with), visual (i.e. transverse taped lines on the floor to step on or over) or tactile (i.e. vibration feedback to step in time with) cues) are recommended physiotherapy strategy for Parkinsonian gait impairment [[8](#_ENREF_8)], which are easily implemented within the home by patients or clinicians.

Physiotherapy guidelines suggest that different cueing strategies (internal or external) may be useful for gait improvement at specific PD stages [[9](#_ENREF_9)] (e.g. H&Y stages I-III). However, cue recommendations for PD are vague (i.e. do not thoroughly differentiate cue effectiveness at PD stages), are based on subjective clinical judgement and are supported by limited objective evidence. Previous cueing studies have largely been conducted in mid-late stage (H&YII-IV) PD participants. Such studies suggest that in the later stages of PD, when FOG and cognitive deficits may be prevalent, only external cues are effective [[10](#_ENREF_10), [11](#_ENREF_11)]. Whereas internal cues are thought to be most useful in early stage PD (H&YI) when cognition is intact. Alternatively, recent evidence has suggested that internal cues may be more effective at improving gait than external cues in mid-late stage PD (H&YII-III) [[12](#_ENREF_12)], which directly contrasts previous physical therapy recommendations. Furthermore, evidence for early PD stage cueing are lacking. A recent pilot study in nine PD participants demonstrated that auditory cue response may alter with PD progression, with limited gait benefits or possible cue induced impairments (e.g. increased variability) in early stage PD (within 6 months of diagnosis: H&YI-II) but increased gait benefits at later stage PD (3 years post diagnosis: H&YII-III) [[13](#_ENREF_13)]. The neural mechanisms underlying gait impairment and cue response in PD are poorly understood, which has led to a lack of knowledge related to cue implementation stage and long-term cue use, as well as reports of variable response [[14](#_ENREF_14)] and selective short-term gait characteristic improvement [[15](#_ENREF_15)].

Previous imaging studies have provided limited indirect evidence of the neural mechanisms involved in gait impairment in PD. Traditional brain imaging techniques, such as functional magnetic resonance imaging (fMRI) positron emission tomography (PET), have shown that the underlying neural mechanisms involved in gait impairment in PD are complex, with gait control relying on contributions from various cortical and sub-cortical centers. For example, studies have highlighted the importance of the pre-frontal cortex (PFC), supplementary motor area (SMA), premotor cortex (PMC), primary motor cortex (M1), basal ganglia, brainstem, cerebellum and spinal cord neural networks in gait control [[16](#_ENREF_16)]. Specifically, within PD there is a shift from automatic to more conscious control of gait in PD, with increased executive-attentional (particularly the frontal and PFC) deployment required to overcome deficits in subcortical regions [[17](#_ENREF_17)]. However, imaging studies are limited to static conditions that primarily use assays of gait (such as virtual reality or mental imagery) to represent task performance [[18](#_ENREF_18)], which limits interpretation of brain activity during actual gait.

Internal and external cue stimulus can improve gait in PD but response is poorly understood, although attentional mechanisms may be key. Theories suggest that different cueing strategies may be underpinned by different attentional mechanisms. External auditory cues are suggested to improve gait through replacement of rhythmic BG output via external stimuli that reduces PFC burden during gait [[19](#_ENREF_19)], and visual or tactile cues may activate attentional mechanisms to allow faster subconscious processing of sensory information when walking [[20](#_ENREF_20), [21](#_ENREF_21)]. Whereas, internal strategies may use attentional mechanisms that by-pass defective basal ganglia (BG) circuitry to improve gait [[22](#_ENREF_22)]. To date however, these theories remain relatively unexplored, likely due to an inability to image the brain during walking. Therefore, specific cortical contributions to different cueing modalities remain unknown and warrant further investigation to inform the most effective strategy to alleviate gait deficit in people with PD.

This will be the first study to investigate comprehensive brain activity during walking and response to internal and external cue stimulus during walking across different PD stages. Recent technological developments have allowed monitoring of brain activity during real-time gait. Progression in mobile brain imaging techniques, such as non-invasive mobile functional near infrared spectroscopy (fNIRS) and electroencephalography (EEG) head caps, have allowed cortical brain activity to be monitored in PD during actual gait, but response to various cueing stimulus across disease stages has yet to be fully determined. Our recent systematic review demonstrated that there is increased activation of specific cortical regions when walking in PD compared to controls, particularly at the PFC [[23](#_ENREF_23)]. However, the majority of previous studies have largely been limited to measurement of only PFC activity due to technology constraints (i.e. fNIRS headbands over the forehead). Indeed, our recent fNIRS headband study showed no change in PFC activity in PD with tactile cues compared to usual walking and no difference between those with or without FOG, which was despite gait improvements with cueing [[24](#_ENREF_24)]. Additionally, EEG studies have shown that those with FOG have increased SMA and frontal cortex activity during FOG episodes when walking, but studies have largely been limited to single EEG channel analysis (i.e. Pz, Cz, Fz or Oz) [[23](#_ENREF_23)], which limits interpretation of specific brain regions. Therefore, brain regional analysis is vital, with previous studies highlighting that cognitive functions are associated with selective gait outcomes in PD [[25](#_ENREF_25)]. For example, attention (involving the PFC) has been related to control of pace-related gait characteristics such as step length and speed, whereas visuo-spatial ability (involving the parietal cortex) has been associated with variability and timing gait characteristics [[25](#_ENREF_25)].

**Study Aims and Hypothesis:**

The overall aim of this research study is to better understand brain activity involved in gait in Parkinson’s disease, gathered during actual walking.

***Aim I: Determine brain activity response to cue stimulus (visual, auditory or tactile) when walking in PD.***

Hypothesis 1a: We hypothesize that internal cueing will demand more PFC activity compared to usual walking and external stimulus strategies, with additional sensorimotor integration required with external cues in those with FOG.

Hypothesis 1b: We hypothesize that external auditory, visual and tactile cueing will be underpinned by selective cognitive, motor and sensorimotor regional brain activation, with more diffuse activation in those with FOG.

***Aim II: Examine changes in brain activity to cues at different stages of PD.***

Hypothesis 2a: We hypothesize that H&YI will respond more to internal than external cue stimulus, but with the least activation of the PFC at this PD stage compared to H&YII and H&YIII.

Hypothesis 2b: We hypothesize that external cue stimulus will elicit multi-region cortical response at H&YIII, with activation of PFC, motor and parietal regions dependent upon cue stimulus type.

**Methods**

*Design*

This is a basic science research study involving human participants. The design of this study will be an exploratory observational study of brain activity during gait in PD, specifically observing brain activity changes with internal (thinking about larger steps) and external (visual, auditory or tactile) cue stimulus while walking.

*Participants*

This study will involve 80 participants with PD, who will be split into groups dependent on the severity of their disease (classified with the Hoehn and Yahr (H&Y) scale); n=20 H&Y stage I (early disease, minimal symptoms); n=30 H&Y stage II (mild disease, no balance issues); n=30 H&Y stage III (moderate disease, balance issues).

As per peer-review amendments, within the H&Y stage II and III groups, we will also ensure recruitment of a sub-group of n=15 individuals who self-report FOG within each group (n=30 total with FOG), which will provide a sub-group for further data analysis. We will limit FOG sub-group recruitment to these groups as we do not expect any individuals with FOG to be in H&Y stage I. Self-reported FOG will be based upon a question in the new Freezing of Gait Questionnaire after seeing the short clip related to the questionnaire. Subjects will be categorized as “freezers” if they have experienced such a feeling or episode over the past month. In addition, we will evaluate the presence of FOG in the laboratory during clinical examination, and if patients are seen with FOG but report 0 on the FOG questionnaire, they will be considered as freezers for the analysis.

*Study Setting*

The setting for the study will be the clinical gait laboratory at the Coach Lane Campus, Northumbria University. The Clinical Gait Laboratory is a dedicated facility for the investigation of gait, balance and mobility in healthy and clinical populations linking with the research themes of the Department of Sport, Exercise and Rehabilitation. The facility has all of the necessary equipment and space to allow this study to be conducted. Qualified physiotherapists will be present during the assessment visits. Reasonable travel expenses will be paid to participants for additional visits to normal care, with reimbursement based on the production of receipts, pre-paid taxi, or mileage allowances as appropriate.

Subjects will be recruited if they meet to the following criteria:

**Inclusion/Exclusion Criteria**

*Inclusion Criteria*

- Clinical diagnosis of Parkinson’s by a movement disorder specialist according to UK brain bank criteria
- H&Y stage I-III
- Aged >50 years
- Able to walk and stand unaided
- Adequate hearing (as evaluated by the whisper test; stand 2m behind subject and whisper a 2 syllable word, subject repeats word) and vision capabilities (as measured using a Snellen chart – 6/18-6/12).
- Stable medication for the past 1 month and anticipated over a period of 6 months

*Exclusion Criteria*

- Psychiatric co-morbidity (e.g. Schizophrenia, major depressive disorder as determined by geriatric depression scale - GDS-15; <10 [[26](#_ENREF_26)])
- Clinical diagnosis of dementia or other severe cognitive impairment (Montreal cognitive assessment <21 [[27](#_ENREF_27)])
- History of neurological disorders other than PD (e.g. Huntington’s disease, stroke, traumatic brain injury, multiple sclerosis, Alzheimer’s disease etc.)
- Acute lower back or lower extremity pain, peripheral neuropathy, rheumatic and orthopaedic diseases
- Unstable medical condition including cardio-vascular issues (e.g. angina, myocardial infarction, pulmonary embolism etc.) in the past 6 months
- Unable to comply with the testing protocol
- Interfering research project or clinical therapy (e.g. currently involved in another clinical trial at the hospital or university involving pharmaceuticals, exercise or any other intervention that may impact their ability to walk)

**Recruitment**

Participants will be recruited from Movement Disorder Clinics at Northumbria Healthcare NHS and Gateshead Health NHS. People with PD will be identified through attendance at the Movement Disorders Clinic at Northumbria Healthcare NHS foundation trust, which are led by Professor Richard Walker (co-investigator of this study). Dr Sam Stuart (PI of this study) attends this clinic weekly to work clinically as an honorary clinical physiotherapist. People with PD will be identified through attendance at the Movement Disorders Clinic at Gateshead Health NHS foundation trust, which are led by Dr Claire McDonald (consultant geriatrician). Verbal consent to their clinician will be required from patients in clinic for their details to be passed to the researchers. Research personnel will be available at clinics as required to invite participants to consider the study, and referrals will be made to the researchers during their attendance (or via secure nhs.net email). If sufficiently interested, participants will be given a Participant Information Sheet (PIS) concerning the study. The invitation will be followed up by a telephone call during the week to assess willingness to participate. If willing, a mutually convenient time for assessment will be organised, and the invitation to attend will be extended to a carer or spouse.

Participants will also be recruited from Parkinson’s UK, we will provide the charity with the lay summary of the study and a participant information sheet, with the contact details of the Principle Investigator. Parkinson’s UK will place this study on their study recruitment section of their website and newsletters to advertise the study to potential participants. Potential participants who get in touch with the principle investigator to be involved in the study will be screened for inclusion and exclusion criteria over the telephone, and if eligible for the study, a mutually convenient time for assessment will be organised, and the invitation to attend will be extended to a carer or spouse.

Participants will also be identified using the DeNDRoN Research Case Register. Registrants have a confirmed diagnosis of dementia or neurodegenerative disease (including Parkinson’s Disease) and have expressed their interest in hearing about local research relevant to their condition. Registrants have given their written or verbal consent for their medical notes to be accessed and screened by members of the DeNDRoN team to match them to a (NIHR portfolio) study. The register currently has several hundred PD patients registered who have expressed their willingness to consider research participation, therefore it is anticipated the register will positively aid recruitment to this study.

Only members of the North East and N. Cumbria DeNDRoN team are permitted access to the register, therefore the initial approach will be made by them. If the patient expresses interest during the initial approach (consisting of a telephone call briefly outlining the study), with their verbal consent, their contact details will be provided to the study team for further follow up.

**Informed consent**

The participant must personally sign and date the latest approved version of the Informed Consent form before any study specific procedures are performed.

Written versions of the Participant information sheet and Informed Consent form will be verbally discussed and presented to the participants detailing no less than: the exact nature of the study; what it will involve for the participant; the implications and constraints of the protocol; any risks involved in taking part. It will be clearly stated that the participant is free to withdraw from the study at any time for any reason without prejudice to future care, without affecting their legal rights, and with no obligation to give the reason for withdrawal.

The participant will then be given time to consider the information, and will have the opportunity to question the Investigator, their GP or other independent parties to decide whether they will participate in the study.

Written Informed Consent will then be obtained by means of participant dated signature and dated signature of the person who presented and obtained the Informed Consent. The person who obtained the consent must be suitably qualified and experienced and have been authorised to do so by the Principal Investigator. A copy of the signed Informed Consent form will be given to the participant. The original signed form will be retained at the study site.

**Testing Protocol**

A repeated measure observational design will be employed with assessments performed during one session (as below) of up to 3 hours. Subjects (n=80) participating in this study will be assigned to groups; H&Y stage I (n=20), or H&Y stage II (n=30) and H&Y stage III (n=30), after an initial screening.

Session 1; (up to 180min)

- Initial screening and clinical assessment (up to 60-90min)
- Brain activity during gait (up to 60-90min)

1. **Initial Screening and Clinical Assessment Protocol**

*Past Medical History Interview*

The principal investigator or one of the investigators acting as his or her representative will provide the participants with the necessary oral and written explanations to obtain their signed/written informed consent before beginning the study.

Each participant will then be interviewed by a member of the study team. The interview will include questions regarding education level, falls history, activity level and side dominance.

*Neuropsychological Tests*

***Montreal cognitive assessment (MoCA)***

Cognitive function will be assessed using standardized neuropsychological tests such as the MoCA; a rapid screening instrument for global cognitive dysfunction [[27](#_ENREF_27)]. Different cognitive domains are assessed (attention and concentration, executive functions, memory, language, visuo-constructional skills, conceptual thinking, calculations, and orientation). The MOCA was found to be a valid instrument for cognitive screening in MCI and PD. In this study the MoCA will be used as a descriptive measure [[27](#_ENREF_27)].

***Geriatric depression scale (GDS-15)***

The short form GDS-15 which was created in 1986 by Sheikh and Yesavage will be used to evaluate subjects depression. This involves 15 questions about the mood of the subjects. Scores of 0 to 4 to be in the normal range, 5 to 9 to indicate mild depression, and 10 to 15 to indicate moderate to severe depression [[26](#_ENREF_26)].

***Attention Computer Battery***

A computerised battery will be used to examine attention, via simple button pressing tasks that measure reaction time, such as simple reaction time, choice reaction time and digit vigilance. The attention battery The attention battery is a valid means of testing attention and has been used in a number of studies involving both PD and cognitively impaired individuals [[28](#_ENREF_28)]. The attention battery involves a series of computerised tests, which the subjects respond to on screen stimuli by pressing one of two buttons.

***Trail Making Test (TMT) Parts A and B***

The Trail Making Test is a visual attention assessment that has been used within various neurological and cognitively impaired groups since the 19040s. The test is done in two parts; A and B. The patient is asked to draw lines to consecutively numbered circles on one work sheet (Part A) and then to connect the same number of consecutively numbered and lettered circles on a separate work sheet, alternating between the two sequences (Part B). Subjects are told to work quickly and not to deviate from the appropriate sequence [[29](#_ENREF_29)]. The examiners test the subjects and record the time, in seconds, needed to complete each part of the test. Any errors are counted and corrected by the examiner, with the timer still going during correction time.

***Benton’s Judgement of Line Orientation Test (JLO)***

The JLO is a high test-retest reliability test and has been shown to have good neuropsychological construct validity via neuroanatomical localization studies [[30](#_ENREF_30)]. JLO is a test of visuospatial ability, which involves a subject viewing a set of numbered lines and then being shown two lines of the same orientation. They then have to name the numbers that the shown lines correspond to.

***Clock copying (e.g. Royall’s CLOX 1 and 2)***

Clock drawing (CLOX 1 and 2) test is a measure of cognitive impairment, which is an internally consistent measure that is easy to administer and has good reliability between raters. Clock drawing is seen as a visuospatial task linked with right parietal pathology. Participants are required to draw a clock with the numbers and arrows pointed at a particular time. Then the subjects have to copy a clock drawn by the researcher [[31](#_ENREF_31)].

*Ophthalmological Tests – basic visual sensory functions*

***Visual acuity (VA) (LogMAR)***

VA is measured binocularly used a standard LogMAR chart. Participants will be seated at a distance of 4m from the chart. Participants will be instructed to read aloud down the chart starting from the top left. All correct answers are recorded on a pre-set score sheet. Test is terminated if the participant makes 2 consecutive errors.

Final LogMAR calculated with the following formula: LogMAR = (score of the line before termination) – (0.02 x number of errors) + (0.02 x correct answers in the terminal line)

***Contrast sensitivity (CS) (Mars letter CS chart, Mars Percetrix™, New York, USA)***

CS will be measured using the Mars CS sheets placed on an adjustable holder. The sheet consists of 48 Latin letters of uniform height; the contrast from the white background decreases with subsequent letters. Room illumination is adjusted so that average CS sheet luminance is between 80 and 120cd/m² (measured via a luminance meter). Assessment is done binocularly with the average distance from the patients eyes being 50cm. Participants read aloud down the sheet starting at the top left. Errors are recorded on the pre-set score sheet and testing is terminated after 2 consecutive errors. Final logCS calculated using the following formula:

LogCS = (value of final correct letter before stopping) – (number of errors prior to stopping x 0.04).

*Disease Specific / Severity Tests*

***The Unified Parkinson's Disease Rating Scale UPDRS-III***

The Unified Parkinson's Disease Rating Scale (motor sub-score) part III, will be used to assess motor and non-motor features of PD and disease severity. The UPDRS is a short clinical assessment of disease severity / motor symptoms; higher scores reflect worsening disability.

***Hoehn & Yahr (H & Y)***

The Hoehn and Yahr rating scale is a widely used clinical rating scale, which defines broad categories of motor function in Parkinson’s disease (PD). All participants’ will be tested who are in H &Y stages I-III.

***The new FOG questionnaire (new FOGQ)***

Freezing of gait (FOG) will be evaluated using the new FOG questionnaire. This is a 10 item questionnaire intended to classify freezing of gait. The questionnaire has 3 parts; distinction of freezers from non-freezers, Freezing severity, frequency and duration and impact of freezing on daily life.

***Falls efficacy scale – International (FES-I)***

Fear of falling will be measured using the falls efficacy scale – international version. This is a short and valid measure of fear of falling in older adults, which assesses basic and demanding activities (both physical and social) [[32](#_ENREF_32)]. It consists of 16 scenarios (e.g. cleaning the house) and subjects must rate their fear of falling on a scale from 1 (Not at all concerned) to 4 (Very concerned).

*Sensorimotor assessment*

**Senaptec Sensory Station**

Sensorimotor skills will be assessed on the computerised Senaptec Sensory Station in sitting or standing. The Senaptec Sensory Station collects and analyses data relating to 10 sensory parameters; visual clarity/ contrast sensitivity/ depth perception/ near-far quickness/ perception span/ multiple object tracking/ reaction time/ target capture/ eye-hand coordination/ go-no-go. This assessment takes ~10minutes in total.

*Orthostatic hypotension*

**Orthostatic Hypotension Questionnaire**

The Orthostatic Hypotension Questionnaire assesses the burden of orthostatic hypotension symptoms. Patients are asked to report, throughout a set of structured questions, symptoms severity and how they impact daily activities. This questionnaire takes approximately 2 minutes to complete.

1. **Brain Activity during Gait Protocol**

Participants (n=80) will be required to walk under different conditions; including but not limited to single task, dual task (e.g. walk while talking) and with a cue stimulus (e.g. thinking about taking larger steps, transverse lines on the floor to step over, metronome beats or vibrating bracelet to step in time with). This will take place in the clinical gait laboratory at coach lane campus, using a testing protocol that has been refined by our team and is applied to all of our gait studies. The different PD participant groups (H&Y 1, 2, 3) will perform the same walking conditions; with repeat measures being taken for an average to be created.

Brain activity will be assessed using a mobile combined functional near infra-red spectroscopy (fNIRS) and electroencephalography (EEG) device (Artinis Medical Systems, The Netherlands), which will be head mounted to each participant using the manufacturers elastic head cap. The fNIRS/EEG device is lightweight (approx. 0.2kg) and consists of fNIRS optode and EEG electrode sensors (on a washable headband) which record data non-invasively from the scalp of participants.

Video recording and small body worn monitors (e.g. accelerometers) will record participant movement during walking and enable specific gait characteristics (e.g. speed, step length etc.), to be identified. Video is used when processing data to check the accuracy of the data and identify causes for potentially anomalous results, such as the participant stopping. Within each trial, data will be averaged across repeated samples and stride pooled for estimation of stride-to-stride fluctuations in gait.

**Primary Outcome Measure**

The primary outcome measure for this study will be brain activity during gait measured by a wireless portable non-invasive fNIRS / EEG system (Artinis Medical Systems, The Netherlands). The fNIRS aspect of the device will quantify changes in cerebral oxy-haemoglobin (HbO_2_). The EEG aspect of the device will quantify electrical activity within the brain, which will be outputted as power spectral densities (PSD), at alpha (8-13Hz), beta (13-30Hz), delta (0.5-4Hz), theta (4-8Hz) and gamma (30-40Hz) frequency bandwidths.

**Secondary Outcome Measure**

Gait characteristics recorded via video recording and wearable sensors (e.g. accelerometers), such as speed and gait variability recorded during different tasks. For example; during walking under different conditions (including but not limited to single and dual task, and with a cue stimulus in place). Spatiotemporal gait characteristics (e.g., gait speed (m/s), variability (CV%), step/stride length (m), stride time (s), swing time (%), asymmetry, and step width (cm)) will be determined.

**Safety Considerations**

There are no major disadvantages or risks in taking part in this study, and there should be no adverse events. All measurements are non-invasive and place the subject at no risk other than those that normally may occur during walking. For some of the patients, fatigue may occur during the testing if they are not used to any kind of physical activity, such as walking. The data collection methods have been developed to minimise the amount of walking, however, to prevent excessive fatigue, subjects will be encouraged to take breaks as needed throughout all study procedures.

It is possible that wearable sensors or the fNIRS/EEG head cap (with electrode gel) may be uncomfortable for some subjects, therefore we will adjust the devices to find the best way to correctly place them (i.e. sensor straps or cap tightness).

The fNIRS/EEG head cap will be cleaned/washed after each participant (we have two headbands to alternate between) to ensure appropriate adherence to infection control policies. Subjects hair will also be washed following assessments; we have accessible shower facilities next to the gait laboratory.

Based on past research experience and on-going clinical experience we do not anticipate that any risks will be involved in the use of a dual-task or cue stimulus in the laboratory setting.

This is an observational study involving assessment. Unrecognized medical issues may be identified during the course of the assessment that require further attention. The investigator performing the assessment will take appropriate action which will usually mean contacting the participant’s GP who can assess matters further.

**COVID-19:** this study will follow government and university guidance for the global pandemic, with risk assessments for these guidelines. We will follow strict guidance on personal protective equipment (PPE) for both participants and researchers, and will be able to socially distance ourselves within the large gait lab space (which has an active ventilation system). The study involves the use of different wearable technologies, which can largely be applied by the participant themselves, without the researcher needing to be in close proximity. Only two researchers will be present during testing to reduce the number of people in the room, and where possible carers or spouses who attend with subjects will be asked to wait outside of the gait lab in our seating area. All participants (and carers or spouses) will be asked if they have had any COVID-19 symptoms within the past several weeks over the phone the day before the visit, and will have their temperature checked upon arrival to the lab for their visit. Researchers will wear masks and gloves during the assessment, and the subjects and researchers will wash their hands upon touching others, or entering or leaving the room (alcohol gel will be available throughout the visit to be used when necessary). The gait laboratory will undergo a ‘deep clean’ for 30 minutes after the participant has attended the laboratory.

**Data Analysis**

Data analysis for fNIRS and EEG signals will be conducted separately but we will compare outcomes from simultaneous recordings from the two systems, which will ensure robust results.

*fNIRS data analysis* will be performed in line with our previous reliable PD fNIRS walking studies [[24](#_ENREF_24), [33](#_ENREF_33)]; 1) HbO_2_ data will be low-pass filtered (cut-off 0.14Hz); 2) Corrected for baseline (removing median of 20sec of initial standing before walks from signal); 3) Reference channel corrected (short 1.5cm channels will be taken away from long 3cm channel signals); 4) Visual signal inspection; and 5) Averaging across fNIRS channels for regions of interest (ROI). A 3D digitizer (Polhemus Patriot) will obtain morphological locations for cortical ROIs relative to scalp position and the fNIRS optodes. Data form the digitizer will be entered into the software package NIRS-statistical package metric mapping (NIRS-SPM, http://www.nitrc.org/projects/nirs_spm), which will be implemented in Matlab. NIRS-SPM allows registration of fNIRS channel data onto the Montreal Neurological Institute (MNI) standard brain space using probabilistic registration of the fNIRS co-ordinate data to determine channels related to ROIs at the group level.

*EEG data analysis* will be performed in line with our previous pilot data analysis, with advice from the developers of the EEGLab analysis software (Professor Scott Makeig, UCSD). Initial signal processing will be conducted using the EEGLab toolbox within Matlab [[34](#_ENREF_34)], which will involve band-pass filtering 1-250Hz and extraction of separate brain and artefact sources in the EEG signals with Independent Component Analysis [[35](#_ENREF_35)]. Source localized independent components (ICs) will be derived and ICs related to brain activity will be identified using the ICLabel function [[36](#_ENREF_36)]. ICs will be clustered (via dipoles and K-means) according to anatomical location, with PSDs extracted from each cluster, including alpha (8-13Hz), beta (13-30Hz), delta (0.5-4Hz), theta (4-8Hz) and gamma (30-40Hz) bandwidths.

**Statistical Analysis**

*Sample Size*

To determine an appropriate sample size we used our previous fNIRS study that examined response to tactile cueing in PD (n=25, H&Y I-III) [[24](#_ENREF_24)]. Given the total effect size of 0.52 for HbO_2_ difference in walking with and without tactile cues, we require at least 18 people per group (H&YI, II, III) for adequate power (α=0.05, 1-β=0.95). Additionally, given the total effect size of 1.43 for mobility differences during tactile cueing between those with (n=25) and without FOG (n=18) in our previous study (Mancini et al. 2018), we require at least 14 people with FOG for adequate power (α=0.05, 1-β=0.95). Considering that we expect larger effect sizes for more severe people with PD and smaller in less severe people with PD, we should be adequately powered with a total sample size of 80, with groups of ≥20 subjects at each H&Y stage (I-III) and for those with FOG (n=30 across H&Y stages II and III).

Initial Analysis

Statistical analysis will be undertaken using SPSS version 25 or more recent versions (SPPS, Inc. an IBM company). All statistical tests will be carried out at the 5% two-sided level of significance. Demographic characteristics and baseline data will be summarized using descriptive statistics, including means, standard deviations, median, minimum, maximum and inter-quartile ranges for continuous or ordinal data and percentages for categorical data. The descriptive statistics will be tabulated and presented graphically for clarity.The assessments recorded at pre-testing will be taken as baseline values.

One-sample Kolmogorov-Smirnov tests will be used to check for normally distributed data. Non-normally distributed continuous distributions will be transformed where appropriate to meet the requirements of parametric tests; otherwise equivalent non-parametric tests will be adopted.

Further Analysis

**Aim I:** ***Determine brain activity response to cue stimulus (visual, auditory or tactile) when walking in PD.***

*Hypothesis 1a:* To test whether increased PFC activation occurs with internal compared to external cues in PD, and whether additional sensorimotor integration is required with external cues in FOG, we will use Linear Mixed Effects Models (LMEMs) to compare PFC and parietal HbO_2_ (fNIRS) and PSD (EEG) values from walking with and without internal and external (auditory, visual, tactile) cues, with FOG status as a between group variable.

*Hypothesis 1b:* To test whether response to external cueing strategies is underpinned by selective cognitive, motor and sensorimotor activity, with more diffuse activity in those with FOG, we will use separate LMEMs to compare regional (entire cortex) HbO_2_ and PSD values from walking with different external cue stimulus (auditory vs visual vs tactile), with FOG status as a between group variable.

LMEMs will have age and gender as covariates, and Bonferroni correction for multiple comparisons.

**Aim II: Examine changes in brain activity response to cue stimulus at different stages of PD.**

*Hypothesis 2a:* To test whether H&YI respond more to internal than external cues with less PFC activity than later PD stages, we will compare PFC HbO_2_ and PSD data, with and without internal and external cues across PD stages (H&YI vs H&YII vs H&YIII). Separate LMEMs will determine how PFC cue stimulus response changes from H&YI to later stages. FOG status, gender and age will be added as model covariates.

*Hypothesis 2b:* To test if brain activity response to cue stimulus becomes more diffuse in later compared to earlier PD, we will compare regional (entire cortex) HbO_2_ and PSD values with and without cues across disease stages (H&YI vs II vs III), and within those who do and do not report FOG. Separate LMEMs will determine how brain activity cue stimulus response changes between PD stages, particularly H&YIII compared to H&YI-II. An additional LMEM will examine cue response between those with (n=30) and without FOG (n=50). Regression analysis will also be performed between brain activity response (regional HbO_2,_ PSD values) to different cues, disease severity (UPDRS-III) and FOGQ score across the cohort (n=80).

LMEMs will have age and gender as covariates, and Bonferroni correction for multiple comparisons.

**Statistical Analysis Interpretation.**

**Aim I:** If HbO_2_ increases and PSD (alpha and beta) values decrease in the PFC in PD during internal compared to external cued walking then our hypothesis that increased attentional activation in PD is required for internal cue response will be confirmed. Additionally, if HbO_2_ increases and PSD (alpha and beta) values decrease in parietal regions with external cues in FOG compared to those without FOG, then our hypothesis that those with FOG require greater sensory integration to respond to external cues will be confirmed. Similarly, if cortical activation is greater in PFC and SMA with auditory cues, but the parietal cortex is more active with visual and tactile cues, with higher activation across regions in those with FOG, this will demonstrate that regional brain activity is responsible for different cue response and more diffuse activation is required in FOG for cue response.

**Aim II:** If brain activity changes more with internal than external cues within H&YI compared to H&YII-III, and there is less increase in HbO_2_ or decrease in PSD at the PFC in H&YI compared to H&YII-III, then our hypothesis that early stage PD respond more to internal cues with less attentional activation needed will be confirmed. Similarly, if H&YIII or those with FOG have greater activation of involved cortical regions (PFC, parietal or SMA) than H&YI-II or those with no-FOG, as well as activation at other brain regions, and higher UPDRS-III correlates to brain activation levels, then our hypothesis that more diffuse brain activity is required for cue stimulus response in later disease and FOG will be confirmed.

**Data Protection and Patient Confidentiality**

The study will comply with the General Data Protection Regulation (GDPR) and Data Protection Act 2018, which require data to be de-identified as soon as it is practical to do so. The processing of the personal data of participants will be minimised by making use of a unique participant study number only on all study documents and any electronic database(s). All data samples collected as part of this study will be anonymised with participants being assigned a unique study number (e.g. PD01, PD02 etc.). All electronically stored data (e.g. videos) will be named using the individuals study number to ensure confidentiality. The only information we will retain for our database will be the age and sex of participants and whether they are a patient. We will keep one hard copy of the assessment in locked filing cabinets in the Clinical Gait Laboratory, Coach Lane, Northumbria University. This is the only place where we store any personal details like names and addresses. This information is kept locked away and is only available to people directly running the study. These people will treat the information in the strictest confidence. Dr Samuel Stuart, the Principle Investigator of this study, is ultimately responsible for the protection of this information.

The Principle Investigator (Dr Stuart) will ensure that this study is conducted in accordance with relevant regulations and with Good Clinical Practice. The results of any tests will be kept strictly confidential. This data can only be accessed directly by the development team of the application, and is securely password protected. The data is kept and stored according to the university's regulations and will be destroyed as such when the study is complete. There is no personal or identifiable data stored in the application itself, as that would be a breach of data security. Information is also kept in accordance to GDPR and will be destroyed according to the appropriate timescales. Once the study has completed its main objectives, data will be stored for 10 years after which it will be disposed of.

**Data Sharing**

The dataset may be used for secondary analysis with the consent of the participants (on consent form), and all participant documentation will reflect the potential future use of these data in research. To maximise impact from this research study, following the end of the study anonymous data will be securely stored on an external online repository, such as FigSHARE with which Northumbria University has an existing partnership. Secondary researchers will be required to reference the data being used.

**Access to Data**

Direct access will be granted to authorised representatives from the Sponsor and host institution for monitoring and/or audit of the study to ensure compliance with regulations. The annonymous dataset may be used for secondary analysis with the consent of the participants (via consent form). All patient documentation will reflect the future use of these data in research.

**Project Management**

*Study Organisation and Management*

The study will be run by Dr Samuel Stuart (Principal Investigator) and researchers within his team. The Northumbria University team will be responsible for ensuring progress of the study in relation to administrative, clinical and academic issues. All published output from the study will acknowledge researchers involved.

**Insurance Indemnity**

The University has a specialist insurance policy in place which would operate in the event of any participant suffering harm as a result of their involvement in the research (U.M. Association Limited)**.**

**Peer Review and Patient and Public Involvement**

This project and protocol design (including cohorts and statistical analysis) has been peer reviewed by three independent experts within the Parkinson’s Foundation Clinical Research Award grant review, with feedback incorporated into the study design and protocol (i.e. additional FOG sub-group included). The protocol has also been internally peer reviewed by the study investigators.

Several people with Parkinson’s and older adults were contacted to provide Patient and Public Involvement (PPI) for this study. The participants, provided valuable feedback on the protocol. Overall, the participants were enthusiastic about the aims of the study, particularly as it related to measurement of cognitive function and motor activity, which the participants recognised were major problems. Further understanding of cueing in relationship to improvement of gait, was also regarded as being a very positive aim.

The participant information sheet (PIS) was considered readable, free of jargon, although repetitive in places, and required a section on legal rights. The PIS has been revised accordingly to make it less repetitive and shorter, and included legal rights as a section.

The participants did not raise any issues regarding design of the research. They were satisfied with the length of the study session and the duration of walking bouts.

### **Dissemination policy**

The data arising from the study is owned by Northumbria University:

On completion of the study, the data will be analysed and tabulated and a Final Study Report prepared.

The final study report can be accessed in the office of the PI, Dr Samuel Stuart.

- All participating investigators have rights to publish any of the study data, with agreement from the other investigators.
- The Parkinson’s Foundation will be acknowledged as the funding body within the study. They have review and publication rights of the data from the study.
- The participants will be notified of the outcome of the study via a specifically designed newsletter.
- Participants can specifically request results which will be provided after the Final Study Report had been compiled.
- The study protocol, full study report, anonymised participant level dataset, and statistical code for generating the results will be made available on request.

**Authorship eligibility guidelines and any intended use of professional writers**

To warrant authorship on publications all study investigators will examined for the following four authorship criteria:

- Substantial contributions to the conception or design of the work; or the acquisition, analysis, or interpretation of data for the work; AND
- Drafting the work or revising it critically for important intellectual content; AND
- Final approval of the version to be published; AND
- Agreement to be accountable for all aspects of the work in ensuring that questions related to the accuracy or integrity of any part of the work are appropriately investigated and resolved

**Work schedule**

The chart below (Table 1, Page 20) shows each study major milestone and the expected time of delivery over the course of the study period. Special attention will be given to the training period on equipment and procedures, which will be critical to test and ameliorate the experimental procedures described above. For example, this training period will be used to define the best placement for fNIRS optodes / EEG electrodes in order to record appropriate brain activity during the walking conditions.

***Table 1. Study major milestones and the expected time of delivery***

| **Milestones** | **Year 1** | | | | | | | | | | | | **Year 2** | | | | | | | | | | | |
| --- | --- | --- | --- | --- | --- | --- | --- | --- | --- | --- | --- | --- | --- | --- | --- | --- | --- | --- | --- | --- | --- | --- | --- | --- |
|  | **1** | **2** | **3** | **4** | **5** | **6** | **7** | **8** | **9** | **10** | **11** | **12** | **13** | **14** | **15** | **16** | **17** | **18** | **19** | **20** | **21** | **22** | **23** | **24** |
| Training (equipment and procedures) | X | X | X |  |  |  |  |  |  |  |  |  |  |  |  |  |  |  |  |  |  |  |  |  |
| Participant recruitment |  | X | X | X | X | X | X | X | X | X | X | X | X | X | X | X | X | X | X | X | X | X |  |  |
| Data collection |  |  | X | X | X | X | X | X | X | X | X | X | X | X | X | X | X | X | X | X | X | X | X |  |
| Data analysis and interpretation |  |  | X | X | X | X | X | X | X | X | X | X | X | X | X | X | X | X | X | X | X | X | X | X |
| Scientific report (for funder / REC) |  |  |  |  |  |  |  |  |  |  |  | X |  |  |  |  |  |  |  |  |  |  |  | X |
| Manuscript preparation/ submission |  |  |  |  |  |  |  |  | X | X | X | X | X | X |  |  |  |  |  |  | X | X | X | X |

***REFERENCES***

1. Muslimović, D., et al., *Determinants of disability and quality of life in mild to moderate Parkinson disease.* Neurology, 2008. **70**(23): p. 2241-2247.

2. Giladi, N., et al., *Freezing of gait in patients with advanced Parkinson's disease.* Journal of Neural Transmission, 2001. **108**(1): p. 53-61.

3. Heremans, E., A. Nieuwboer, and S. Vercruysse, *Freezing of gait in Parkinson’s disease: where are we now?* Current neurology and neuroscience reports, 2013. **13**(6): p. 350.

4. Sethi, K., *Levodopa unresponsive symptoms in Parkinson disease.* Movement disorders, 2008. **23 Suppl 3**: p. S521-33.

5. Curtze, C., et al., *Levodopa Is a Double-Edged Sword for Balance and Gait in People With Parkinson's Disease.* Mov Disord, 2015. **30**(10): p. 1361-70.

6. Henderson, E.J., et al., *Rivastigmine for gait stability in patients with Parkinson's disease (ReSPonD): a randomised, double-blind, placebo-controlled, phase 2 trial.* The Lancet Neurology, 2016. **15**(3): p. 249-258.

7. Munoz-Hellin, E., R. Cano-de-la-Cuerda, and J.C. Miangolarra-Page, *Visual cues as a therapeutic tool in Parkinson's disease. A systematic review.* Rev Esp Geriatr Gerontol, 2013. **48**(4): p. 190-7.

8. Keus, S.H.J., et al., *Evidence-based analysis of physical therapy in Parkinson's disease with recommendations for practice and research.* Movement Disorders, 2007. **22**(4): p. 451-460.

9. Rochester, L., S. Lord, and M.E. Morris, *The role of physiotherapy in the rehabilitation of people with movement disorders*, in *Rehabilitation in Movement Disorders*, M.E. Morris and R. Iansek, Editors. 2013, Cambridge University Press: Cambridge. p. 55-68.

10. Gräber, S., et al., *Post-cueing deficits with maintained cueing benefits in patients with Parkinson's disease dementia.* Frontiers in neurology, 2014. **5**: p. 236-236.

11. Rochester, L., et al., *Evidence for motor learning in Parkinson's disease: Acquisition, automaticity and retention of cued gait performance after training with external rhythmical cues.* Brain Research, 2010. **1319**: p. 103-111.

12. Harrison, E.C., A.P. Horin, and G.M. Earhart, *Internal cueing improves gait more than external cueing in healthy adults and people with Parkinson disease.* Scientific Reports, 2018. **8**(1): p. 15525.

13. Lirani-Silva, E., et al., *Auditory Cueing for Gait Impairment in Persons With Parkinson Disease: A Pilot Study of Changes in Response With Disease Progression.* Journal of Neurologic Physical Therapy, 2019. **43**(1): p. 50-55.

14. Suteerawattananon, M., et al., *Effects of visual and auditory cues on gait in individuals with Parkinson's disease.* J Neurol Sci, 2004. **219**(1-2): p. 63-9.

15. Morris, M.E., C.L. Martin, and M.L. Schenkman, *Striding out with Parkinson disease: evidence-based physical therapy for gait disorders.* Phys Ther, 2010. **90**(2): p. 280-8.

16. Zwergal, A., et al., *Aging of human supraspinal locomotor and postural control in fMRI.* Neurobiol Aging, 2012. **33**(6): p. 1073-84.

17. Nantel, J., et al., *Deficits in visuospatial processing contribute to quantitative measures of freezing of gait in Parkinson's disease.* Neuroscience, 2012. **221**: p. 151-6.

18. Gilat, M., et al., *Brain activation underlying turning in Parkinson's disease patients with and without freezing of gait: a virtual reality fMRI study.* NPJ Parkinsons Dis, 2015. **1**: p. 15020.

19. Cunnington, R., R. Iansek, and J.L. Bradshaw, *Movement-related potentials in Parkinson's disease: external cues and attentional strategies.* Mov Disord, 1999. **14**(1): p. 63-8.

20. Johansson, R. and G. Westling, *Roles of glabrous skin receptors and sensorimotor memory in automatic control of precision grip when lifting rougher or more slippery objects.* Experimental brain research, 1984. **56**(3): p. 550-564.

21. Rabin, E. and A.M. Gordon, *Prior experience and current goals affect muscle-spindle and tactile integration.* Experimental brain research, 2006. **169**(3): p. 407-416.

22. Morris, M.E., et al., *Stride length regulation in Parkinson's disease. Normalization strategies and underlying mechanisms.* Brain, 1996. **119 ( Pt 2)**: p. 551-68.

23. Stuart, S., et al., *Cortical activity during walking and balance tasks in older adults and in people with Parkinson’s disease: A structured review.* Maturitas, 2018. **113**: p. 53-72.

24. Stuart, S. and M. Mancini, *Prefrontal Cortical Activation With Open and Closed-Loop Tactile Cueing When Walking and Turning in Parkinson Disease: A Pilot Study.* Journal of Neurologic Physical Therapy, 2019. **Publish Ahead of Print**.

25. Morris, R., et al., *Gait and cognition: Mapping the global and discrete relationships in ageing and neurodegenerative disease.* Neurosci Biobehav Rev, 2016. **64**: p. 326-45.

26. Aikman, G.G.a.O., M.E. , *Geriatric Depression Scale: Long Form Versus Short Form.* Clinical Gerontologist, 2000. **22**(3/4): p. 63-70.

27. Dalrymple-Alford, J.C., et al., *The MoCA: Well-suited screen for cognitive impairment in Parkinson disease.* Neurology, 2010. **75**: p. 1717-1725.

28. Wesnes, K.A., et al., *Benefits of rivastigmine on attention in dementia associated with Parkinson disease.* NEUROLOGY, 2005. **65**: p. 1654–1656.

29. Fals-Stewart, W., *An interrater reliability study of the Trail Making Test (Parts A and B).* Perceptual and Motor Skills, 1992. **74**(1): p. 39-42.

30. Calamia, M., et al., *Developing a short form of Benton's Judgment of Line Orientation Test: an item response theory approach.* Clin Neuropsychol, 2011. **25**(4): p. 670-84.

31. Royall, D.R., J.A. Cordes, and M. Polk, *CLOX: an executive clock drawing task.* J Neurol Neurosurg Psychiatry, 1998. **64**: p. 588-594.

32. Yardley, L., et al., *Development and initial validation of the Falls Efficacy Scale-International (FES-I).* Age Ageing, 2005. **34**(6): p. 614-9.

33. Stuart, S., et al., *Pre-frontal Cortical Activity During Walking and Turning Is Reliable and Differentiates Across Young, Older Adults and People With Parkinson's Disease.* Frontiers in Neurology, 2019. **10**(536).

34. Delorme, A. and S. Makeig, *EEGLAB: an open source toolbox for analysis of single-trial EEG dynamics including independent component analysis.* Journal of neuroscience methods, 2004. **134**(1): p. 9-21.

35. Makeig, S., et al. *Independent component analysis of electroencephalographic data*. in *Advances in neural information processing systems*. 1996.

36. Pion-Tonachini, L., K. Kreutz-Delgado, and S. Makeig, *ICLabel: An automated electroencephalographic independent component classifier, dataset, and website.* NeuroImage, 2019. **198**: p. 181-197.
