# Supplementary material for: Brain activity response to cues during gait in Parkinson’s disease: A study protocol
Source: PLoS One. 2022 Nov 17;17(11):e0275894. doi: 10.1371/journal.pone.0275894 (PMC9671304; doi:10.1371/journal.pone.0275894)
Supplement: Attachment — Submitted filename: PONE-D-21-34386.docx [file pone.0275894.s003.docx]

The manuscript “**Brain activity response to cues during gait in Parkinson's disease: a study protocol**” by Stuart *et.al.* deals with the concurrent assessment of gait and brain cortical responses using different cueing strategies. The research findings will provide support to more tailored approaches for the application of cues to improve gait in Parkinson's disease. These findings should thus motivate further research and would deserve publication.

I am positive about this work and would suggest this work for publication.
