# Supplementary material for: Brain activity response to cues during gait in Parkinson’s disease: A study protocol
Source: PLoS One. 2022 Nov 17;17(11):e0275894. doi: 10.1371/journal.pone.0275894 (PMC9671304; doi:10.1371/journal.pone.0275894)
Supplement: Attachment — Submitted filename: PONE-D-21-34386_Response to reviewers.docx [file pone.0275894.s004.docx]

Dear Dr. Parasuraman Padmanabhan,

Thank you for the opportunity to revise and resubmit the manuscript. Of course, many thanks to the reviewers for taking the time to comment on the manuscript and for providing feedback, which is greatly appreciated. Reviewers’ comments and suggestions helped us to clarify additional aspects in the manuscript. Our specific responses are listed below.

We have highlighted the changes in the document by using the Track Changes tool. A clean copy of our revised manuscript is also included in the resubmission.

**Reviewer #1**

***The manuscript “Brain activity response to cues during gait in Parkinson's disease: a study protocol” by Stuart et.al. deals with the concurrent assessment of gait and brain cortical responses using different cueing strategies. The research findings will provide support to more tailored approaches for the application of cues to improve gait in Parkinson's disease. These findings should thus motivate further research and would deserve publication.***

***I am positive about this work and would suggest this work for publication.***

We appreciate reviewer’s comments and recommendation for publication.

**Reviewer #2**

***1. What type of sampling was adopted in choosing the subjects***

No specific type of sampling is adopted. As described in the manuscript, patients with a confirmed diagnosis of Parkinson’s disease are informed about the study and those who agree to learn more about it are contacted by a member of the research team who explains the study further. If sufficiently interested, patients are invited and given a Participant Information Sheet concerning the study. The invitation is followed up by a telephone call to assess willingness to participate. If willing, a mutually convenient time for the study visit is organised.

***2. Statistical analysis of results if possible can be provided, that will improve the impact of the work***

Please note that this paper is submitted as a “Study Protocol” and, therefore, does not include data/results. A paper describing the observed effects on brain cortical activity and gait parameters is planned for later this year (or early next year).

***3. Hybrid brain imaging modalities are taken, please highlight the cons, while using these modalities individually***

EEG and fNIRS quantify different physiological processes related to brain activity. EEG measures the electromagnetic field created when neurons in the brain fire; and fNIRS measures the hemodynamic response, which is the adjustment of oxygen in the blood when a brain region is active. The obvious con of using one of these modalities individually would be the restriction to one physiological process. However, we believe that the discussion of cons regarding the use of a specific brain imaging modality is beyond the scope of our protocol paper and, therefore, we have not added this discussion to the paper.

***4. Future work can be highlighted in the discussion section***

Thank you for your suggestion. We have added the following paragraph to the Discussion section: “*Findings have potential to support the development of future research aiming to maximize the response to cueing in PD. Cortical regions responsive to cueing may be targeted by other interventions, such as non-invasive brain stimulation, to enhance benefits from cueing. Thus, the development of combined interventions (e.g., non-invasive brain stimulation + cueing) would be a reasonable next step to be explored. To date, transcranial direct current stimulation has been shown to enhance the effects of other combined interventions, such as exercise or cognitive training, in people with PD (47, 48).*”

***5. Recent papers can be included in the introduction session***

Thank you for your suggestion. We have added citations to two recent review papers on cueing in PD:

- Forte et al., 2021: [https://pubmed.ncbi.nlm.nih.gov/34067458/#](https://pubmed.ncbi.nlm.nih.gov/34067458/)

- Gonçalves et al., 2021: [https://pubmed.ncbi.nlm.nih.gov/33969461/#](https://pubmed.ncbi.nlm.nih.gov/33969461/)

***6. Whether this hybrid medical imaging modality (fNIRs+EEG)is applicable for other neuro disorder analysis***

Thank you for your suggestion. Although we are confident that hybrid fNIRS + EEG is applicable for other clinical populations, this aspect is beyond the scope of our Study Protocol and, therefore, we have not added such information/discussion to the manuscript.
